# Insulin enhanced leptin-induced STAT3 signaling by inducing GRP78

**DOI:** 10.1038/srep34312

**Published:** 2016-09-28

**Authors:** Mina Thon, Toru Hosoi, Koichiro Ozawa

**Affiliations:** 1 Department of Pharmacotherapy, Graduate School of Biomedical and Health Sciences, Hiroshima University, 1-2-3 Kasumi, Minami-ku, Hiroshima 734-8551, Japan

## Abstract

Leptin, an adipocyte-derived hormone, centrally regulates energy homeostasis. Overlaps in the regulation of glucose and energy homeostasis have been reported between leptin and insulin. However, the effects of insulin on leptin’s actions in the central nervous system (CNS) have not yet been elucidated in detail. In the present study, we found that insulin potentiated leptin’s actions through GRP78 in the neuronal cell line, SH-SY5Y-ObRb. Since insulin induces GRP78, we speculated that it may also enhance leptin’s actions through this induction. We found that insulin enhanced leptin-induced STAT3 phosphorylation and this effect was ameliorated by the knockdown of GRP78. The role of GRP78 in leptin’s actions was also confirmed by impairments in leptin-induced STAT3 phosphorylation in HEK293-ObRb cells in which GRP78 was knocked down. Furthermore, we found that the overexpression of GRP78 enhanced leptin-induced STAT3 phosphorylation. These results suggest that GRP78 plays an important role in leptin’s actions. Furthermore, insulin may enhance the leptin-induced activation of STAT3 by inducing GRP78, which may provide an important connection between insulin and leptin in the CNS.

Over the last few decades, the incidence of obesity has been increasing at a rapid rate^1^,^2^. Obesity and its associated complications eventually lead to a number of metabolic disorders such as diabetes, hypertension, hyperlipidemia, nonalcoholic fatty liver disease, and cardiovascular diseases^3^. Leptin, an anti-obesity hormone secreted from adipocytes, plays critical roles in the regulation of appetite and feeding-related behavior. Leptin’s main anti-obesity signaling pathway is predominantly involved in its binding to the Ob-Rb long isoform of the leptin receptor. The neuronal response to leptin receptor activation is mediated through the Janus tyrosine kinase (JAK)-signal transducer and activator of transcription (STAT) cascade^4^,^5^,^6^. It currently remains unknown whether leptin is the only hormone involved in the control of energy balance. Besides its peripheral actions, the pancreatic hormone, insulin, mediates the hypothalamic regulation of energy homeostasis and glucose metabolism^7^,^8^. This hypothalamic regulation of insulin is basically mediated through the insulin receptor substrate (IRS)-phosphatidylinositol-3-kinase (PI3K) pathway^9^,^10^.

Although leptin and insulin signal transduction pathways are distinct, they both act on the same hypothalamic area in order to decrease eating behavior^11^. The deletion of insulin receptors in neurons has been shown to result in obesity in mice^11^; however, this effect was milder than that of leptin^12^. Recent evidence suggests crosstalk between leptin and insulin, particularly in the regulation of food intake^13^. A previous study revealed that the co-infusion of leptin and insulin potentiated leptin-induced JAK2-STAT3 signal transduction^13^.

In eukaryotic cells, most proteins are translocated into the endoplasmic reticulum (ER) for subsequent folding and quality-control checks prior to reaching their final destination^14^. Therefore, the ER is an important organelle for cell survival and maintaining proper cellular functions. Unfolded or misfolded proteins accumulate in the ER when its function is impaired, which causes ER stress. As an adaptive response, cells resist stress by activating the unfolded protein response (UPR). UPR functions to restore normal cell function by attenuating protein translation in order to reduce the loading of unfolded proteins in the ER through the promotion of protein folding and activation of degradation pathways^15^,^16^,^17^. Glucose-regulated protein 78 (GRP78), a major up-regulated UPR chaperone located in the lumen of the ER, is an important protein that is required to maintain ER capacity and protect against ER stress by assisting in protein folding, which prevents aggregation^14^,^18^,^19^. Therefore, the induction of GRP78 appears to be critical for maintaining normal cell functions, thereby alleviating ER stress and ER stress-related diseases.

ER stress has been associated with neurodegenerative disorders^20^,^21^, diabetes^22^, and cancer^23^. We and others previously reported the involvement of ER stress in leptin resistance^24^,^25^,^26^, a hallmark of obesity^27^. Based on the induction of GRP78 by insulin^28^ and its critical role in response to ER stress, the aim of the present study was to investigate the function of GRP78 in leptin signaling in insulin-treated neuronal cells.

## Results

### Insulin induced the phosphorylation of Akt and S6K

**Figure 1:**
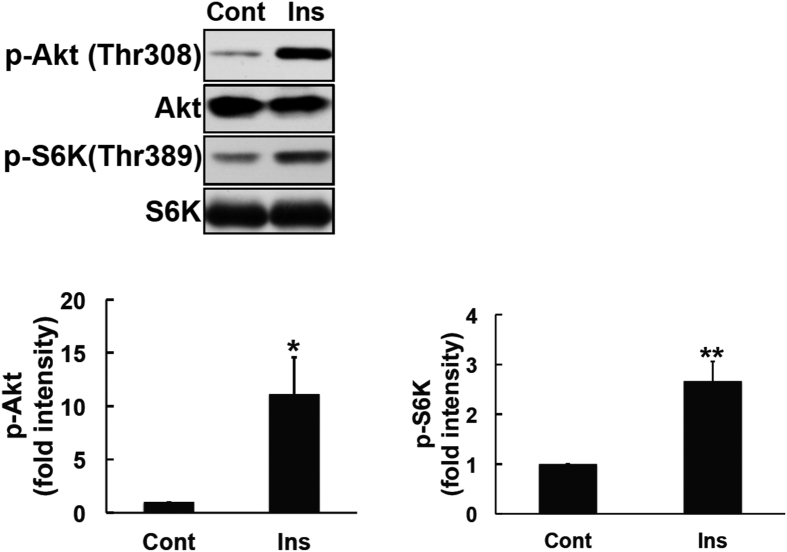
Insulin induced Akt and S6K activation in SH-SY5Y-ObRb cells. SH-SY5Y-ObRb cells were incubated in serum-free medium for 20 h and treated with insulin (300 nM) for 4 h. Phospho-Akt (Thr308), Akt, phospho-S6K (Thr389), and S6K were analyzed by Western blotting. Insulin increased the phosphorylation of Akt (Thr308) and S6K (Thr389) in SH-SY5Y-ObRb cells. A densitometric analysis of phospho-Akt (Thr308) and phospho-S6K (Thr389) was performed using image analysis software. Data are expressed as the mean ± S.E. of 4 independent experiments (n = 4). *P < 0.05; **P < 0.01.

PI3K and mammalian target of rapamycin (mTOR) activities are necessary for insulin-induced metabolic pathways^29^,^30^. The activation of these pathways results in the phosphorylation of its downstream proteins Akt^31^ and p70 ribosomal S6 kinase (S6K)^32^. A human neuroblastoma cell line stably transfected with the Ob-Rb leptin receptor (SH-SY5Y-ObRb) was treated with insulin (300 nM, 4 h). As shown in Fig. 1, insulin markedly activated and phosphorylated Akt and S6K in SH-SY5Y-ObRb cells. Thus, insulin signaling was functionally activated in the SH-SY5Y-ObRb cell line.

Additionally, to check that insulin-induced signal would be functional in the ObRb receptor stably transfected SH-SY5Y cells, we analyzed insulin-induced Akt phosphorylation in both SH-SY5Yand SH-SY5Y-ObRb cell lines. SH-SY5Y and SH-SY5Y-ObRb cells were exposed to serum-free medium for 24 h. Insulin (10 and 100 nM) was added during the last 15 min. Insulin-induced Akt activation signal was then analyzed by Western blotting. The result showed that insulin induced Akt phosphorylation in both models (Supplementary Figs 1 and 2).

### Insulin enhanced leptin-induced STAT3 activation

**Figure 2:**
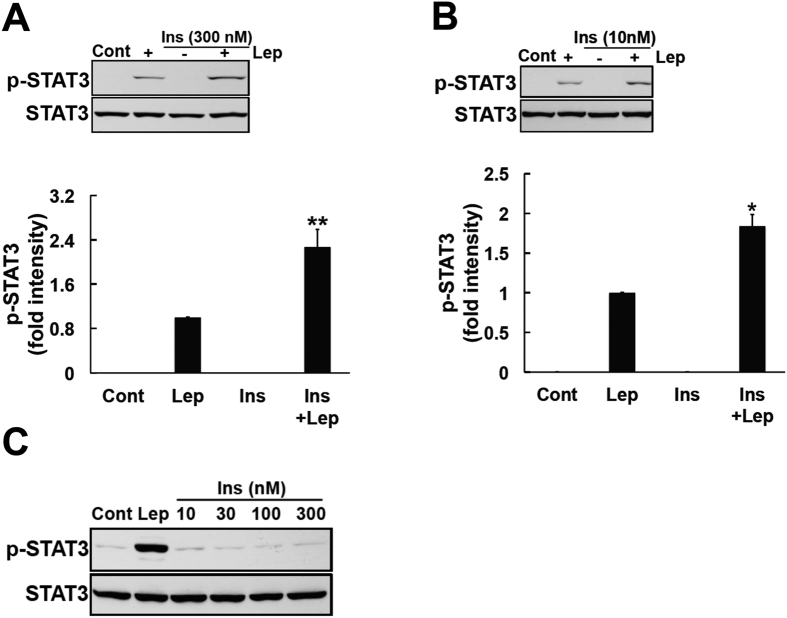
Insulin enhanced leptin-induced STAT3 phosphorylation in SH-SY5Y-ObRb cells. (**A)** SH-SY5Y-ObRb cells were incubated in serum-free medium for 20 h. Cells were treated with insulin (300 nM) for 4 h, followed by leptin (0.03 μg/ml, 15 min). Phospho-STAT3 (Tyr705) and STAT3 levels were analyzed by Western blotting. A co-stimulation with insulin and leptin enhanced the leptin-induced phosphorylation of STAT3. (**A**) densitometric analysis of phospho-STAT3 (Tyr705) was performed using image analysis software. Data are expressed as the mean ± S.E. of 4 independent experiments (n = 4). **P < 0.01. (**B)** SH-SY5Y-ObRb cells were incubated in serum-free medium for 20 h. Cells were treated with insulin (10 nM) for 4 h, followed by leptin (0.03 μg/ml, 15 min). Phospho-STAT3 (Tyr705) and STAT3 levels were analyzed by Western blotting. Pretreatment with insulin enhanced the leptin-induced phosphorylation of STAT3. A densitometric analysis of phospho-STAT3 (Tyr705) was performed using image analysis software. Data are expressed as the mean ± S.E. of 3 independent experiments (n = 3). *P < 0.05. (**C**) SH-SY5Y-ObRb cells were incubated in serum-free medium for 24 h. Cells were then treated with insulin (10, 30, 100 and 300 nM). Leptin (0.03 μg/ml, 15 min) was used as a positive control. After 15 min, phospho-STAT3 (Tyr705) and STAT3 levels were analyzed by Western blotting. Insulin did not induce the phosphorylation of STAT3 in SH-SY5Y-ObRb cells. Typical data of 3 independent experiments were shown.

By binding to the long isoform of its receptor, leptin plays a major role in preventing obesity by activating the JAK2-STAT3 signaling pathway^4^,^5^,^6^. Insulin and leptin target the same hypothalamic area in order to suppress eating behavior^11^; therefore, an investigation of the interaction between leptin and insulin may provide a fundamental understanding of obesity and its related diseases. In the present study, we examined the effects of leptin in the presence or absence of insulin using SH-SY5Y-ObRb cells. In order to achieve this, medium was changed to a serum-free condition 20 h prior to the exposure of cells to insulin (10 and 300 nM, 4 h) and this was followed by a leptin stimulation (0.03 μg/ml, 15 min). Consistent with previous findings^33^, we showed that the co-stimulation with insulin and leptin enhanced leptin induced-STAT3 more than the leptin treatment alone (Fig. 2A,B).

At the same time, we also investigated the effect of insulin alone on STAT3 phosphorylation. SH-SY5Y-ObRb cells were exposed to serum-free medium for 24 h. Various concentration of insulin (10, 30, 100 and 300 nM) was added during the last 15 min. As a result, solely treatment with insulin did not induce phosphorylation of STAT3, even at a short time (Fig. 2C).

### Involvement of GRP78 in insulin-induced enhancements in leptin’s actions

**Figure 3:**
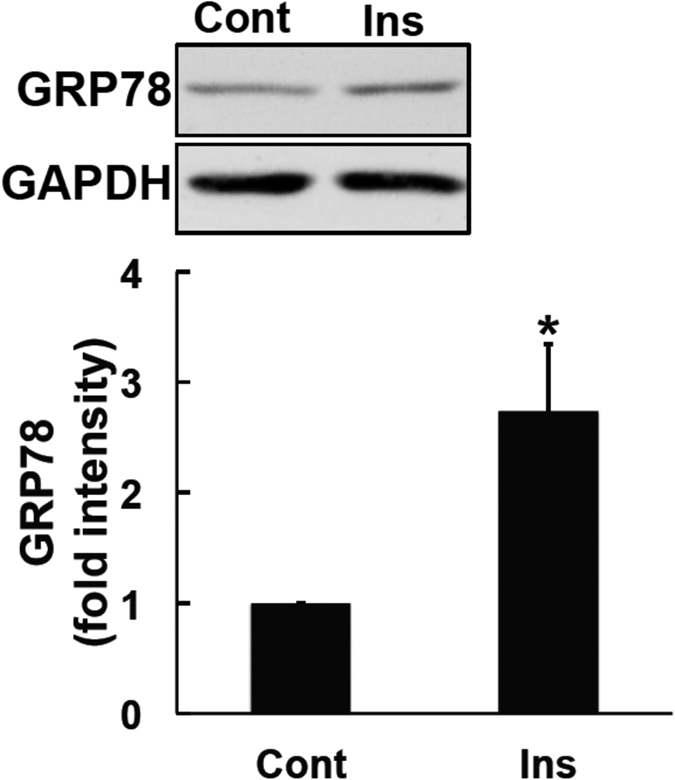
Insulin induced GRP78 expression in SH-SY5Y-ObRb cells. SH-SY5Y-ObRb cells were incubated in serum-free medium for 20 h. Cells were then treated with insulin (300 nM) for 4 h. GRP78 and GAPDH levels were analyzed by Western blotting. Insulin up-regulated GRP78 levels. A densitometric analysis of GRP78 was performed using image analysis software. Data are expressed as the mean ± S.E. of 4 independent experiments (n = 4). *P < 0.05.

Increasing evidence supports the overlapping roles of leptin and insulin in signal transduction. Leptin and insulin both activate PI3K^13^,^34^. The PI3K-mTOR pathway is one of the upstream pathways involved in the induction of GRP78^35^. Of note, insulin was previously reported to increase GRP78 levels via the PI3K-ATF4 pathway^28^. These findings suggest that insulin-induced GRP78 may influence the anti-obesity effects of leptin. Therefore, we examined the induction of GRP78 by insulin under our experimental conditions. SH-SY5Y-ObRb cells were exposed to serum-free medium for 20 h. Insulin (300 nM) was then added for 4 h. In accordance with previous findings^28^, we showed that insulin induced GRP78 in the SH-SY5Y-ObRb cell line (Fig. 3).

**Figure 4:**
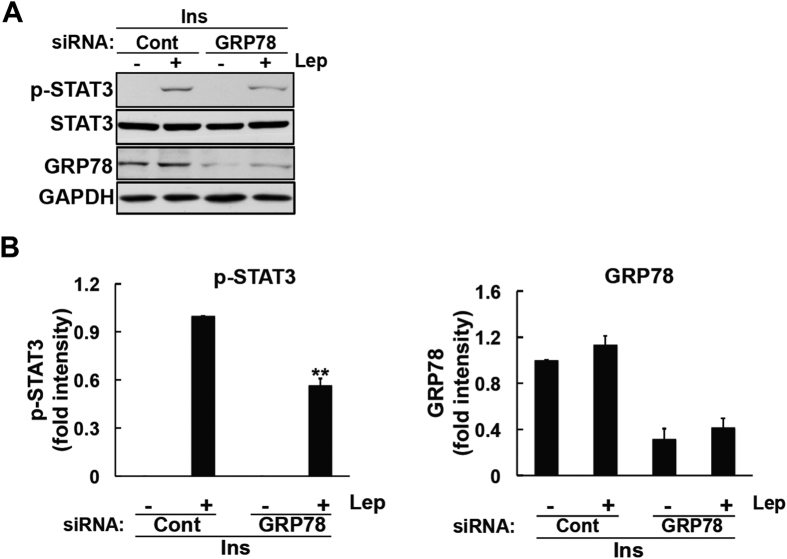
Insulin-mediated enhancements in STAT3 signaling were mediated through GRP78 in SH-SY5Y-ObRb cells. SH-SY5Y-ObRb cells were transfected with siRNA (5 nM) for 72 h. Seventy-two hours after the transfection, medium was changed to serum-free medium for another 20 h. Cells were treated with insulin (300 nM) for 4 h followed by leptin (0.03 μg/ml, 15 min). Phospho-STAT3 (Tyr705), STAT3, GRP78, and GAPDH levels were analyzed by Western blotting. **(A**) Leptin-induced STAT3 phosphorylation in insulin-treated cells was ameliorated in GRP78-knocked down cells. (**B)** A densitometric analysis of phospho-STAT3 (Tyr705) and GRP78 was performed using image analysis software. Data are expressed as the mean ± S.E. of 4 independent experiments (n = 4). **P < 0.01.

We next assessed the influence of insulin-induced GRP78 on leptin signals. We determined whether insulin-induced GRP78 is involved in enhancing leptin-induced STAT3 activation. In order to achieve this, we knocked down GRP78 in insulin-treated cells and analyzed leptin-induced STAT3 phosphorylation. Insulin failed to enhance leptin-induced STAT3 activation in GRP78-knocked down cells (Fig. 4). Taken together, these results suggest that insulin enhances leptin-induced STAT3 phosphorylation via the induction of GRP78.

### Influence of GRP78 in leptin-induced STAT3 phosphorylation

**Figure 5:**
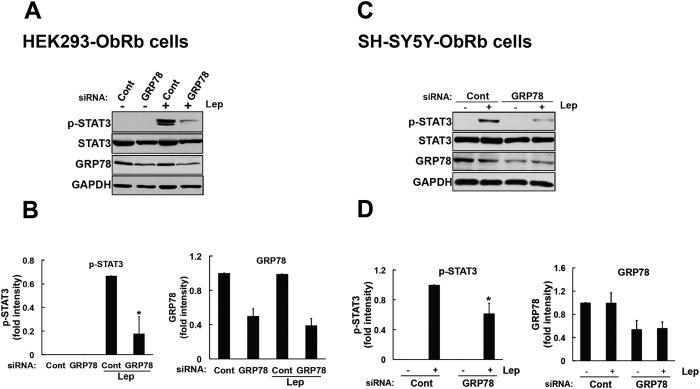
Leptin-induced STAT3 phosphorylation was inhibited by the knockdown of GRP78 in HEK293-ObRb and SH-SY5Y-ObRb cells. HEK293-ObRb cells were transfected with siRNA (2 nM) for 72 h. Seventy-two hours after the transfection, cells were treated with leptin (0.5 μg/ml) for 15 min. Phospho-STAT3 (Tyr705), STAT3, GRP78, and GAPDH levels were analyzed by Western blotting. (**A**) Leptin-induced STAT3 phosphorylation was inhibited in GRP78-knocked down cells. (**B)** A densitometric analysis of phospho-STAT3 (Tyr705) and GRP78 was performed using image analysis software. Data are expressed as the mean ± S.E. of 4 independent experiments (n = 4). *P < 0.05. (**C)** SH-SY5Y-ObRb cells were transfected with control or GRP78 siRNAs (5 nM) for 72 h. Seventy-two hours after the transfection, medium was changed to serum-free medium for another 24 h. Cells were treated with leptin (0.03 μg/ml, 15 min). Phospho-STAT3 (Tyr705), STAT3, GRP78, and GAPDH levels were analyzed by Western blotting. Leptin-induced STAT3 phosphorylation was attenuated in GRP78-knocked down cells. (**D)** A densitometric analysis of phospho-STAT3 (Tyr705) and GRP78 was performed using image analysis software. Data are expressed as the mean ± S.E. of 4 independent experiments (n = 4). *P < 0.05.

We subsequently investigated the role of GRP78 in leptin-induced STAT3 activation. We performed GRP78 knockdown experiments in HEK293-ObRb cells. Cells were transfected with GRP78 siRNA (2 nM) for 72 h. As an indicator of transfection efficacy, the expression of GRP78 was subjected to a Western blotting analysis. We confirmed that GRP78-specific siRNA efficiently inhibited its expression in HEK293-ObRb cells (Fig. 5A,B). Under these conditions, we analyzed leptin-induced STAT3 signaling. We found that the knockdown of GRP78 significantly reduced the leptin-induced phosphorylation of STAT3 in the HEK293-ObRb cell line (Fig. 5A,B).

We simultaneously examined the effect of GRP78 knockdown on leptin-induced signal in SH-SY5Y-ObRb cellular model. SH-SY5Y-ObRb cells were transfected with GRP78 siRNA (5 nM) for 72 h. Seventy-two hours after the transfection, medium was switched to serum-free medium for another 24 h followed by leptin stimulation (0.03 μg/ml, 15 min). GRP78 expression was inhibited by GRP78-specific siRNA (Fig. 5C,D). Within this condition, leptin-induced STAT3 phosphorylation was significantly attenuated in SH-SY5Y-ObRb cells (Fig. 5C,D).

**Figure 6:**
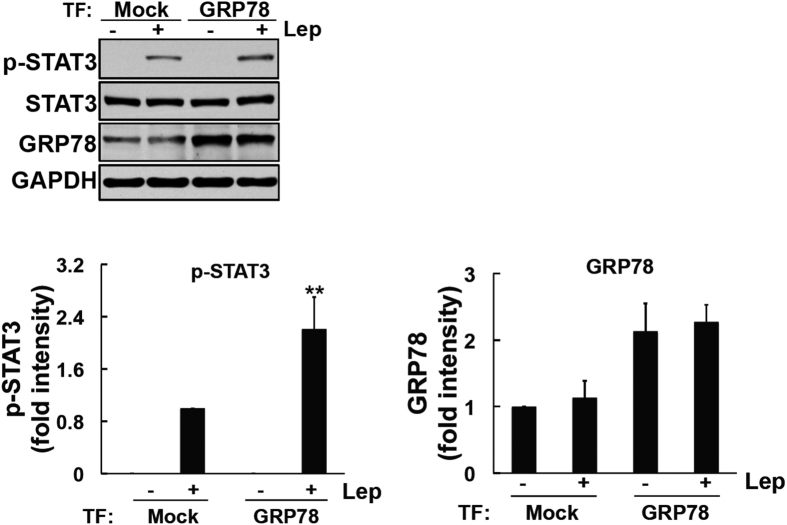
Overexpression of GRP78 enhanced leptin-induced STAT3 phosphorylation in HEK293-ObRb cells. HEK293-ObRb cells were transfected with a GRP78 construct. Forty-eight hours after the transfection, cells were stimulated with leptin (0.03 μg/ml) for 15 min. Phospho-STAT3 (Tyr705), STAT3, GRP78, and GAPDH levels were analyzed by Western blotting. (**A**) GRP78 construct enhanced leptin-induced STAT3 activation. (**B)** A densitometric analysis of phospho-STAT3 (Tyr705) and GRP78 was performed using image analysis software. Data are expressed as the mean ± S.E. of 4 independent experiments (n = 4). **P < 0.01.

In order to elucidate the role of GRP78 in leptin-induced STAT3 signaling, we then overexpressed GRP78 and analyzed leptin-induced signaling. The GRP78 construct was transfected into HEK293-ObRb cells for 48 h and the expression of GRP78 was then analyzed. We confirmed that the transfection of GRP78 increased its levels in HEK293-ObRb cells (Fig. 6). We subsequently stimulated this cellular model with leptin and analyzed STAT3 phosphorylation. As shown in Fig. 6, STAT3 phosphorylation was significantly greater in cells overexpressing GRP78 than in mock-transfected cells. Therefore, these results suggest that GRP78 plays an important role in the activation of leptin-induced STAT3 signaling.

## Discussion

The adipocyte-derived hormone, leptin, and pancreatic hormone, insulin, centrally regulate nutrient homeostasis by suppressing feeding desire^7^,^36^. To date, studies on the relationship between leptin and insulin have been more focused on energy homeostasis^13^. Insulin was previously shown to potentiate leptin-induced STAT3 phosphorylation^33^. Furthermore, it was reported to induce GRP78 and protect against cell death^28^. Although a relationship between insulin and leptin has already been reported^13^,^37^, the function of GRP78 in leptin signaling has not yet been clarified. In the present study, we investigated the function of GRP78 in leptin-induced signaling, and found that it plays an important role in the leptin-induced phosphorylation of STAT3.

Due to the abundant expression of their receptors in the hypothalamus, leptin and insulin exert parallel effects on reductions in food intake and the activation of adiposity signaling. Leptin and insulin were shown to be secreted in a manner that depended on body fat content^38^,^39^. Furthermore, the central administration of either peptide is known to reduce appetite^7^,^40^. Leptin and insulin share the same PI3K molecular pathway. In addition to the JAK2-STAT3 pathway, the PI3K pathway is one of the main pathways involved in the anti-obesity effects of leptin^34^,^41^. The intracerebroventricular administration of a PI3K inhibitor was previously reported to inhibit the effects of leptin on anorexia^42^. Furthermore, insulin inhibited feeding through IRS-PI3K signaling^9^. Another study demonstrated that insulin up-regulated GRP78 through the PI3K-ATF4 pathway in neuronal cells^28^. In the present study, we found that insulin induced the expression of GRP78, while the inhibition of GRP78 expression ameliorated insulin-induced enhancements in leptin-induced STAT3 phosphorylation. Taken together, these results suggest the following link between insulin and leptin: by inducing GRP78, insulin has the ability to enhance leptin-induced STAT3 signaling. Additionally, we found that the knockdown of GRP78 inhibited leptin-induced STAT3 phosphorylation, and leptin-induced STAT3 phosphorylation was enhanced by its overexpression. Therefore, GRP78 may play an important role in leptin-induced STAT3 phosphorylation. Since our current *in vitro* results are interesting, further studies should be conducted in more physiological model, i.e. differentiated model of SH-SY5Y or primary hypothalamic neuronal cells.

Resistance to the effects of leptin is a characteristic of obese patients^27^. However, the pathophysiological mechanisms responsible for leptin resistance have not yet been determined. The accumulation of saturated fatty acids in obesity has been shown to cause ER stress, and one of the mechanisms responsible for leptin resistance may be mediated through ER stress^24^,^26^,^43^. A previous study reported that insulin contributed to resistance against ER stress^28^. Therefore, it may induce GRP78, thereby ameliorating ER stress-induced leptin resistance in obesity. We previously reported that leptin induced GRP78 expression in SH-SY5Y-ObRb cell line^44^. It is interesting subject to analyze whether leptin will affect insulin signaling through GRP78 expression. Further studies are needed in order to elucidate these mechanisms in more detail.

In conclusion, we herein demonstrated the important role of GRP78 in potentiating leptin signaling in a human neuroblastoma cellular model. Our results highlight the significance of GRP78 and its underlying connection to leptin and insulin. This integration may be advantageous for understanding the regulation of energy homeostasis, which may provide useful information for therapeutic interventions for obesity.

## Methods

### Materials

Human leptin was obtained from Enzo Life Science (NY). Human insulin (Humulin^®^R) was purchased from Eli Lilly and Company (IN).

### Cell Culture

Human neuroblastoma (SH-SY5Y) and human HEK293 cell lines stably transfected with the Ob-Rb long form of the leptin receptor (SH-SY5Y-ObRb and HEK293-ObRb, respectively)^45^ were maintained in Dulbecco’s modified Eagle’s medium (DMEM) supplemented with 10% (v/v) heat-inactivated fetal calf serum at 37 °C in humidified 5% CO_2_/95% air. All experiments were performed in DMEM.

### RNAi Experiment

The transient transfection of siRNA was conducted in SH-SY5Y-ObRb cells and HEK293-ObRb cells. Lipofectamine RNAiMAX (Life Technologies) was used to transfect siRNA according to the manufacturer’s guidelines. Opti-MEM medium was used for transfections. We used the following siRNA in the RNAi experiments: Silencer^®^ Select Pre-designed (Inventoried) siRNA (Life Technologies, siRNA ID for GRP78: s6980) and Silencer^®^ Select Negative Control siRNA #1 (Life Technologies). The final concentrations of siRNA were 2 and 5 nM. Cells were harvested 72–96 h after transfections.

### Plasmid and transfections

pcDNA3.1(+)-GRP78/Bip was a gift from Richard C. Austin (Addgene plasmid # 32701)^46^. A mock plasmid was used as a negative control. Human HEK293-ObRb cells were transfected using the calcium phosphate transfection method. A total of 0.5 μg of DNA was used per 35-mm plate. 2xHEPES buffered saline (2xHBS) solution (16.4 g NaCl, 11.9 g HEPES, and 0.54 g Na_2_HPO_4_, per liter, at pH 7.15) was used to form precipitates of calcium phosphate/DNA. Briefly, 3 h prior to transfection, we changed to fresh medium and a solution containing the plasmid and 2.5 mM CaCl_2_ was added to 2xHBS solution. Following a 30-min incubation at room temperature, the calcium phosphate-plasmid suspension solution was added dropwise to the culture dish. Eight hours after the incubation, culture medium was changed to DMEM containing antibiotics. Cells were harvested 48 h after transfections.

### Western Blotting Analysis

Western blotting was performed as described previously^5^,^45^. Cells were washed with ice-cold phosphate-buffered saline and lysed in buffer containing 10 mM HEPES-NaOH, pH 7.5, 150 mM NaCl, 1 mM EGTA, 1 mM Na_3_VO_4_, 10 mM NaF, 10 μg/ml aprotinin, 10 μg/ml leupeptin, 1 mM phenylmethylsulfonyl fluoride, and 1% Nonidet P-40 for 20 min. Lysates were centrifuged at 15,000 rpm at 4 °C for 20 min, and supernatants were collected. Samples were boiled with Laemmli buffer for 3 min, fractionated using SDS-polyacrylamide gel electrophoresis, and transferred at 4 °C to nitrocellulose membranes. Five-percent skimmed milk was prepared in 20 mM Tris-HCl (pH 7.6), 137 mM NaCl, and 0.1% Tween 20 (TBST) buffer, and was then used to block membranes at room temperature for 1 h. The membranes were then incubated with rabbit anti-phospho-Akt (Thr308) (#13038P, Cell Signaling), rabbit anti-AKT (#9272, Cell Signaling), rabbit anti-GRP78 (GL-19) (G8918, Sigma-Aldrich), mouse anti-GAPDH (ACR001P, Acris), rabbit anti-phospho-STAT3 (Tyr705) (#9145S, Cell Signaling), rabbit anti-STAT3 (C-20) (sc-482, Santa Cruz), rabbit anti-phospho-S6K (Thr389) (#9234, Cell Signaling), and rabbit anti-S6K (#2708S, Cell Signaling) antibodies, and this was followed by an incubation with an anti-horseradish peroxidase-linked antibody. Peroxidase was detected using enhanced chemiluminescence with an ECL system (Amersham).

### Statistics

A one-way analysis of variance analysis was used with Bonferroni’s post hoc analysis for comparison between multiple groups. A paired *t*-test was used for comparison between two groups. Significance was defined as a *P* value.

## Additional Information

**How to cite this article**: Thon, M. *et al.* Insulin enhanced leptin-induced STAT3 signaling by inducing GRP78. *Sci. Rep.* **6**, 34312; doi: 10.1038/srep34312 (2016).

## Supplementary Material

Supplementary Information
