# Supplementary material for: Insulin enhanced leptin-induced STAT3 signaling by inducing GRP78
Source: Sci Rep. 2016 Sep 28;6:34312. doi: 10.1038/srep34312 (PMC5039638; doi:10.1038/srep34312)
Supplement: Supplementary Information [file srep34312-s1.pdf]

Supplementary Information

Insulin enhanced leptin-induced STAT3 signaling by inducing GRP78

Mina Thon, Toru Hosoi, Koichiro Ozawa

Department of Pharmacotherapy, Graduate School of Biomedical and Health Sciences, Hiroshima University, 1-2-3 Kasumi, Minami-ku, Hiroshima 734-8551, Japan

*Correspondence to: Koichiro Ozawa (e-mail: ozawak@hiroshima-u.ac.jp) or Toru Hosoi (e-mail: toruh@hiroshima-u.ac.jp), Department of Pharmacotherapy, Graduate School of Biomedical and Health Sciences, Hiroshima University, 1-2-3 Kasumi, Minami-ku, Hiroshima 734-8551, Japan. TEL.: +81-82-257-5338

Supplementary information includes:

Supplementary Figures S1 to S2

Supplementary Figure 1.

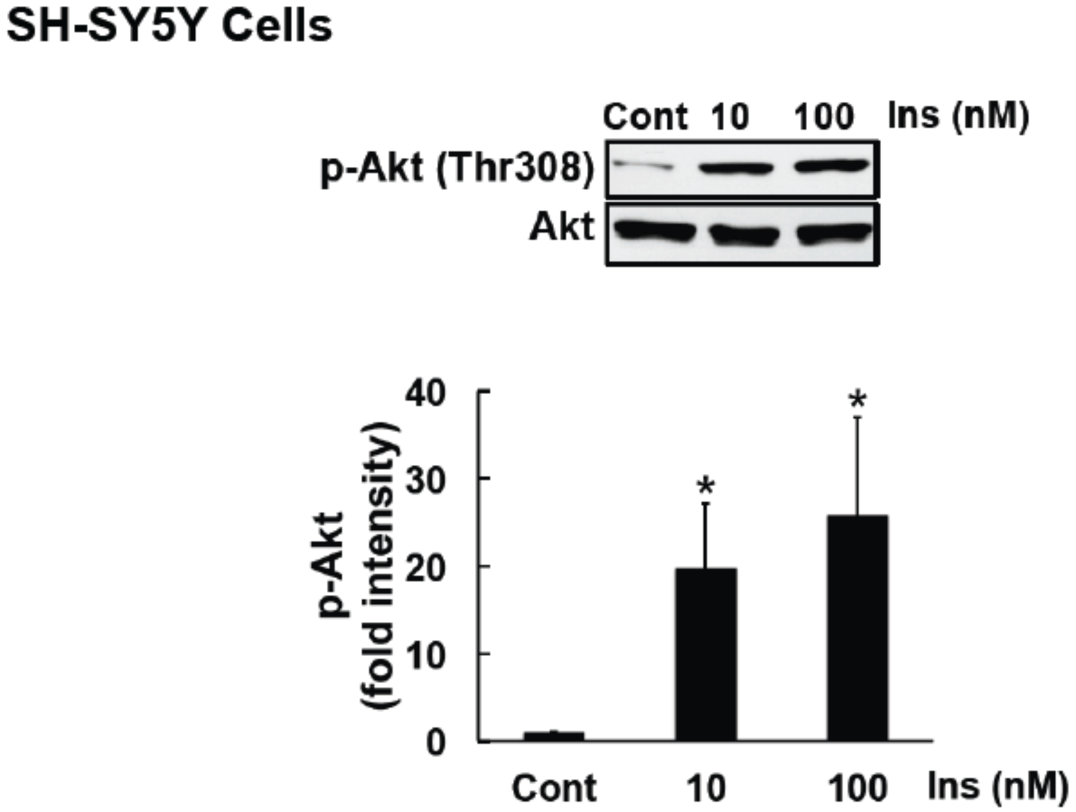

Supplementary Figure 1. Insulin induced Akt activation in SH-SY5Y cells.

SH-SY5Y cells were incubated in serum-free medium for 24 h. Cells were then treated with insulin (10 and 100 nM) for 15 min. Akt phosphorylation levels were analyzed by Western blotting. Insulin up-regulated the phosphorylation of Akt (Thr308) in SH-SY5Y cells. A densitometric analysis of phospho-Akt (Thr308) was performed using image analysis software. Data are expressed as the mean ± S.E. of 3 independent experiments (n=3). *$P < 0.05$.

Supplementary Figure 2.

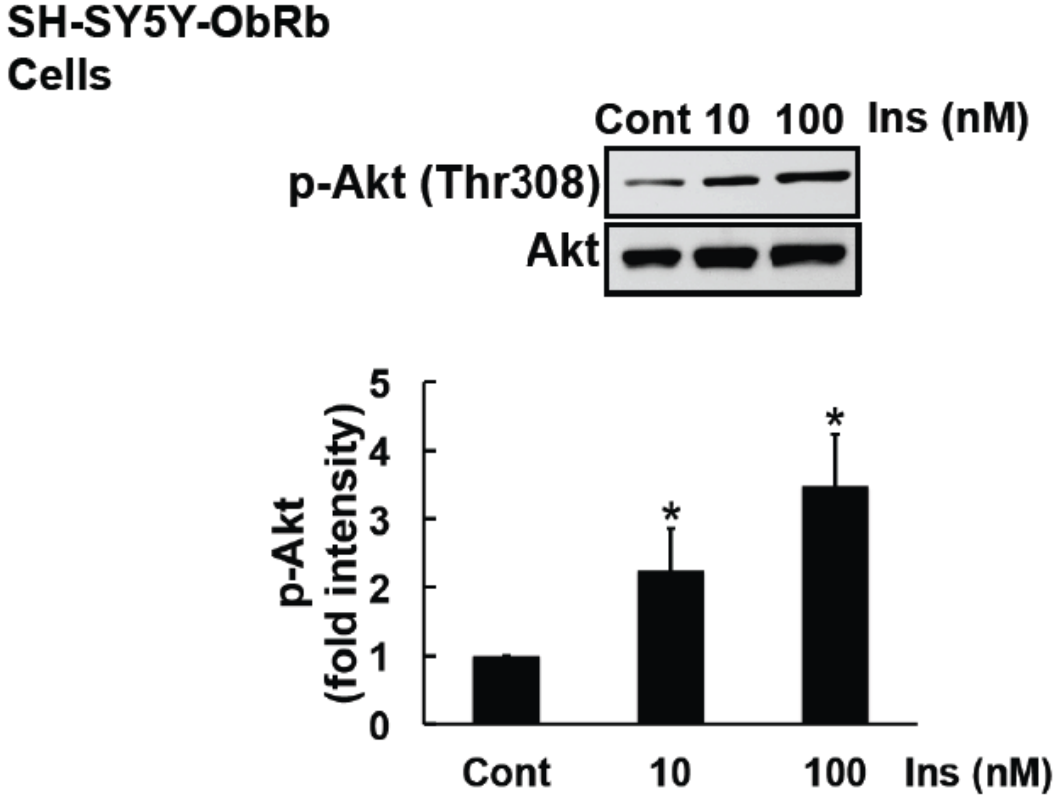

Supplementary Figure 2. Insulin induced Akt activation in SH-SY5Y-ObRb cells.

SH-SY5Y-ObRb cells were incubated in serum-free medium for 24 h. Cells were then treated with insulin (10 and 100 nM) for 15 min. Akt phosphorylation levels were analyzed by Western blotting. Insulin up-regulated the phosphorylation of Akt (Thr308) in SH-SY5Y-ObRb cells. A densitometric analysis of phospho-Akt (Thr308) was performed using image analysis software. Data are expressed as the mean ± S.E. of 3 independent experiments (n=3). *$P < 0.05$.
